# Formulations for Bacteriophage Therapy and the Potential Uses of Immobilization

**DOI:** 10.3390/ph14040359

**Published:** 2021-04-13

**Authors:** Daniel Rosner, Jason Clark

**Affiliations:** Fixed Phage Ltd., Glasgow G20 0SP, UK; jason.clark@fixed-phage.com

**Keywords:** phage therapy, phage formulations, immobilization, encapsulation, therapeutic delivery

## Abstract

The emergence of antibiotic-resistant pathogens is becoming increasingly problematic in the treatment of bacterial diseases. This has led to bacteriophages receiving increased attention as an alternative form of treatment. Phages are effective at targeting and killing bacterial strains of interest and have yielded encouraging results when administered as part of a tailored treatment to severely ill patients as a last resort. Despite this, success in clinical trials has not always been as forthcoming, with several high-profile trials failing to demonstrate the efficacy of phage preparations in curing diseases of interest. Whilst this may be in part due to reasons surrounding poor phage selection and a lack of understanding of the underlying disease, there is growing consensus that future success in clinical trials will depend on effective delivery of phage therapeutics to the area of infection. This can be achieved using bacteriophage formulations instead of purely liquid preparations. Several encapsulation-based strategies can be applied to produce phage formulations and encouraging results have been observed with respect to efficacy as well as long term phage stability. Immobilization-based approaches have generally been neglected for the production of phage therapeutics but could also offer a viable alternative.

## 1. Introduction

It is estimated that antibiotic-resistant bacterial strains account for approximately 33,000 annual deaths in Europe, emphasizing the urgent need for devising novel strategies to tackle this global challenge [1]. The ability of bacteria to acquire drug resistance through random mutation as well as conjugation-mediated genetic transfer has made bacterial infection and contamination a major concern with far reaching implications. *Pseudomonas aeruginosa* strains have been shown to become resistant to colistin through cross-species plasmid transfer of the MCR-1 gene from resistant strains of *Escherichia coli* [2]. Other examples of antibiotic-resistant superbugs include methicillin-resistant *Staphylococcus aureus*, vancomycin-resistant *Enterococcus* (VRE) and multi-drug-resistant *Mycobacterium tuberculosis* [3]. The spread of resistance has resulted in the emergence of ‘superbugs’, responsible for an increase in deaths from illnesses previously treatable with conventional antibiotics [4,5,6,7,8].

The search for alternative strategies to solve these problems has rekindled interest in bacteriophages. These viruses can kill specific bacterial targets, leaving other cells unharmed. Additionally, their ability to propagate to high concentrations at the site of infection reduces the need for continuous application [9,10]. A considerable proportion of bacteriophage-related research now focuses on their practical application for the treatment of diseases including respiratory, gastro-intestinal, wound and skin infections [11,12,13,14,15,16]. While phages can be applied to areas of infection in liquid form, this does not necessarily represent the most effective means of treatment, with the few controlled clinical trials that have been carried out yielding mixed results thus far [17,18,19]. Apart from the difficulty in applying a liquid preparation to a site of infection, adverse conditions brought about by the body’s natural physio-chemical environment as well as its immune response could present a considerable challenge to bacteriophage stability [20]. There has therefore been increased attention towards the development of alternative phage formulations, with a view to improving both their efficiency of application as well as their long-term stability [21,22]. 

The incorporation of bacteriophages into therapeutic formulations typically involves encapsulating them within a stabilizing substance [23,24]. Through such an approach, various antimicrobial materials such as powders, semisolids and nanofibers can be produced, providing more options for effective delivery at the site of infection and, consequently, improved patient outcomes. To this end, encouraging in vitro and in vivo results have been reported for various encapsulated phage formulations including spray and freeze-dried powders, emulsions and liposomes [25,26,27,28]. A strategy that has received considerably less attention with respect to therapeutic formulations is immobilization. Here, phages are instead bound to substrate surfaces. Whilst more commonly applied to the incorporation of bacteriophages into pathogen biosensors, immobilization represents a broad array of techniques which could potentially also be applied in this area. These are discussed in detail in this review, in addition to an overview of the formulation approaches carried out with respect to phage therapeutics thus far. 

## 2. Stabilization and Formulation of Bacteriophage Therapeutics

As with other protein-based macromolecules, bacteriophages are prone to the effects of protein mis-folding and aggregation as well as denaturization, resulting in subsequent loss of functionality when exposed to adverse conditions [29]. Previous studies have reported on the sensitivity of phages to organic solvents, pH, temperature, and salinity [30,31,32,33,34,35]. Several protocols for the long-term storage of free phage have been established by researchers. In general, phages which commonly exist at ambient temperatures can be stored at 4 °C for extended periods of time with only limited drops in titer observed in most cases [36,37]. It should be noted that survivability is highly variable across bacteriophages, with cases of titers depleting over relatively short amounts of time, even when stored at 4 °C [37]. Additional preservation is often observed by freezing at −80 °C [35]. In cases where quick degradation is observed, stability outcomes can be improved using additives such as gelatine, magnesium ions and glycerol [38]. 

The preparation of bacteriophage formulations for therapeutic delivery presents additional challenges compared to the storage of free phage lysates in the lab. Unlike the latter, which are stored long term in favorable conditions, phage formulations may ultimately be subjected to extreme conditions which will vary depending on their application. In the case of gastrointestinal infections, for example, a therapeutic phage cocktail needs to survive and carry out its function in a highly acidic environment which could prove to be too adverse for non-formulated phages. Phages existing in dried, non-liquid formulations are generally more stable in the longer-term but are still affected by thermal and other stresses, which can produce a drop in titer. Additionally, the actual process by which a given formulation is produced can result in bacteriophage degradation, as exemplified by the processes of freeze-drying and spray-drying [39,40]. All these factors are considerations in developing stable phage formulations with the development typically focusing on assessing phage delivery to target bacteria, establishing the extent of the stability a given formulation provides in a range of conditions and improving phage survival during the formulation production process. 

Common methods used to produce phage formulations typically rely on some form of encapsulation. This is a broad term that is used to describe various techniques including emulsification, freeze-drying, spray-drying, liposome encapsulation and electrospinning, in which bacteriophages are coated/surrounded by certain stabilising agents, providing protection against the external environment (Table 1). Once encapsulated phages need to be released from the material to target bacterial cells. 

As discussed previously, the motivation behind investigating alternative formulations for bacteriophages as opposed to pure lysates revolves around the flexibility this can provide clinicians to deliver phages more effectively at the site of infection and improve patient outcomes. In a localized skin infection, for example, several delivery options are made possible. Emulsion encapsulation would allow for the production of a topical cream to be applied directly at the site of infection [44,45]. Freeze-drying and spray-drying techniques can be used to produce phage-coated powders, which can then also be incorporated into a cream for direct application, pill-form for oral application as well as incorporation into an inhaler system [12,22]. Immobilization could be utilized to produce phage-coated patches for direct application onto the skin (bacteriophage immobilization is discussed in more detail later). Additionally, there is also the option of applying the original liquid lysates as either oral drops or as part of a parenteral treatment. Formulations can facilitate viral preservation for longer periods of time in harsher conditions, which facilitates their therapeutic application. A good formulation will also allow for the production of product at large scale in the knowledge that it can be stored easily with minimal periodical drop in phage titer (Table 2). 

### 2.1. Emulsification

Emulsions are mixtures of immiscible liquids in which one liquid acts as the continuous phase with the other(s) dispersed within it with the help of a surfactant. In the case of oil-in-water emulsions, this results in their characteristic cream-like nature, a property that makes the resulting formulation appropriate for topical treatments [45]. Emulsions are thermodynamically stable and are used across various industries including food, health care and chemical synthesis [46]. They can be classified according to the droplet size of the dispersed phase, as well as their thermostability. In conventional emulsions, these measure between 100 nm and 100 µm in diameter and are considered thermodynamically unstable [47]. Micro-emulsions, on the other hand, fall in the 2–50 nm range and are thermodynamically stable [48]. Nano-emulsions represent a different class of emulsion characterised by non-thermodynamically stable droplets (they are, instead, kinetically stable) of diameters measuring 100 nm and below. They are formed through mechanical shear processes [49]. Emulsion-based formulations can be modified to promote percutaneous absorption by varying droplet size, changing the emollient and/or emulsifier and incorporating particulate components into the mixture [50]. This can make them particularly effective in treating deep-rooted infections of the skin.

The dispersal and/or encapsulation of bacteriophages in emulsions has been shown to improve their stability whilst facilitating their bioactivity [51,52]. Esteban et al. (2014) used nano-emulsion encapsulation to stabilize phage K lysates for subsequent in vitro testing against 3 strains of *Staphylococcus aureus* [27]. Nano-emulsions were prepared from a soybean oil—SM buffer mixture using phase inversion temperature. The resultant droplets were 17 nm in diameter, with the authors concluding that the phages were surrounded by nano-droplets as opposed to being encapsulated inside them. Phages in emulsion demonstrated higher activity than their non-emulsified counterparts over a 10-day period at both 4 °C and room temperature. Furthermore, the phages in emulsion were shown to be effective at killing the three *S. aureus* strains, resulting in complete clearance in 2–5 h depending on the strain used. This was notably better than the liquid phage control, in which bacterial growth did eventually resume following an initial clearance of two of the strains. The compounds used to make up the phases of an emulsion can directly affect stability of the phage inside it. Dini et al. (2012) compared the ability of aqueous phases prepared from 2% sodium alginate and 3% low methoxylated pectin to stabilize two *E. coli* 0157:H7 phages in microemulsions containing a 10 % (*v*/*v*) oleic acid oil phase [53]. Emulsions were prepared using agitation. There were notable differences between the two aqueous phases, with low methoxylated pectin—oleic acid emulsions proving to be more effective than those containing sodium alginate at stabilising the phages against acidity and high ionic strength. On the other hand, a lower starting number of phages was recorded within the emulsion droplets for the former, suggesting that the pectin may have interacted with the phages to hinder the encapsulation process. 

More recent work has focused on understanding the ways in which emulsions can facilitate bacteriophage infectivity. Esteban et al. (2018) found that in nano-emulsion encapsulation, there are typically many more droplets than bacteriophages present within the mixture [54]. The authors postulate that both phages and any bacteria within the emulsion are covered in droplets resulting in electrostatic force shielding, in which the negative charges present across bacterial and phage particles are reduced. This, in turn, would act to reduce repulsion between the particles, facilitating phage adsorption and subsequent infection. 

Despite the improved bioavailability and delivery of emulsified bacteriophages, there are challenges to the use of such a technique in an upscaled scenario. Limited work demonstrating long-term viability of phages in this type of formulation has been reported. Cold storage as well as further processing, such as freeze drying of emulsions could be used to increase formulation shelf-life, however this would significantly increase the cost of production. Furthermore, the relatively fragile nature of semi-solid materials as well as the increased risk of bacterial contamination makes them a less attractive option with respect to bulk storage and transport [55]. 

### 2.2. Freeze-Drying

In freeze-drying (lyophilization) the phase change process of sublimation is used to remove all traces of water from a sample [56]. This is achieved by heating up frozen material below the triple point of water (6.12 mbar and 0 °C). In practise, optimal temperature and pressure fall well below the triple point of water, which corresponds to pure water, as opposed to water surrounding and within a material sample together with any additives used. Ice present begins to sublime, resulting in removal of water from the system. Initial freezing is usually carried out by slowly lowering the temperature. This promotes the formation of larger ice crystals which sublime more readily. Larger crystals can alternatively be produced through annealing. To initiate drying, the pressure of the system is lowered below the triple point through formation of a vacuum. Heat is then added gradually, allowing sublimation to occur. The lyophilization process can take several days to complete. The final product is typically a powder of varying grain sizes. This type of formulation can therefore be applied to a range of delivery scenarios, such as incorporation into oral capsules or topical creams. Inhalable freeze-dried powders containing phage cocktails are also being investigated as an alternative treatment for respiratory illnesses [57]. 

Freeze-drying has been carried out extensively on non-phage viruses and more recently been the subject of increased interest for use in bacteriophage stabilisation [57,58,59,60,61]. As with other viruses, the freeze-drying process can be detrimental to bacteriophages. The sudden change of state coupled with the formation of ice crystals challenges the integrity of the phage capsid and can result in large scale loss of phage viability. For this reason, excipients are usually added to the phage solution prior to lyophilization, resulting in the viruses ultimately becoming encapsulated and stabilized within them. Zhang et al. (2018) carried out a study comparing trehalose, mannitol, PEG6000 and sucrose as excipients for the lyophilization of M13 bacteriophage [41]. All 4 compounds were effective in stabilising the phages during the process, following which negligible titer losses were observed, supporting prior findings concerning the cryoprotective properties of sugars and polymers. From the results, it was concluded that phages were stabilized more effectively by the two disaccharides, which were able to preserve phages in dried powder over the long term at ambient temperature (not more than 1 log drop over 2 months). This agrees with previous findings showing trehalose and sucrose work well as excipients [62,63]. 

Proteins have also shown promise for stabilising phages during the freeze-drying process [58]. Liang et al. (2020) showed that *Campylobacter*-targeting bacteriophage CP30A can be lyophilized using tryptone as an excipient with less than a log drop in titer after the process [58]. Larger drops were however recorded following long-term storage in non-refrigerated conditions. Additionally, increased humidity in dried powder adversely affected phage viability. Bacteriophage structure can also influence stability during lyophilization. In studies on a range of animal viruses, Melanovska et al. (2014) concluded that while enveloped virions survived the process in the presence of excipients such as sucrose, gelatine, skimmed milk and sodium glutamate, their non-enveloped counterparts were able to remain viable in culture medium alone [64]. 

The powder-form product of the freeze-drying process makes it an interesting option with respect to large scale production. Powders are generally light and stable, making them easy to pack and transport. Additionally, the potential for freeze dried phage formulations to retain viability over periods of months would allow for a non-disruptive incorporation into a production chain. These advantages need to be considered against the high costs and long waiting times (between 20 and 40 h per freeze drying freeze-drying cycle) associated with the process.

### 2.3. Spray-Drying 

Spray-drying allows for the transformation of a liquid substance to a dried particulate form through evaporation [65]. This is achieved by spraying concentrated feed droplets (typically between 10–100 µm in diameter) into a hot drying chamber containing hot air. A higher substance concentration facilitates the evaporation by reducing the amount of liquid needing to be removed, as does the spraying of atomized particles due to the increased surface area to volume ratio. Once inside the drying chamber, moisture begins to evaporate until a dried shell of the substance of interest remains. An attractive aspect of spray-drying is the relative simplicity of the process, and for this reason, the technique has received increased attention with respect to bacteriophage formulations. The resulting product of the spray-drying process is a dry powder which much like the product of the freeze-drying process, can be applied to creams, tablets and inhalable formulations [57]. 

The drying temperature, air flow rate, type of atomizer as well as droplet size can all influence the extent to which phages survive the process and the phage titer achieved for final product [65]. In particular, the drying temperature typically used for spray-drying, which often exceeds 60 °C, can be especially detrimental to phage viability during the processing [35]. The use of lower drying temperatures set to 50 °C or lower is therefore advisable where bacteriophages formulations are concerned. As with freeze-drying, encapsulation in excipients allows for stabilization of phages during and after the process [66]. Trehalose was used in conjunction with trileucine and pullalan by Carrigy et al. (2020) to stabilize bacteriophage CPA30 for spray-drying, with only a 0.6 log drop in titer being recorded following 1 month of storage at ambient room temperature [67]. Stability over 1 year was demonstrated by Leung et al. (2020) who used varying amounts of trehalose and leucine to stabilize the *Pseudomonas* phages PEV2 and PEV40 during spray-drying [39]. Less than a 1 log drop in titer was observed after the process. The same was true for all formulations, where despite slight differences, all managed to remain within a log of the initial phage load after 1 year at 4 °C and 20 °C in a vacuum.

The advantages with respect to storage, transport and stability associated with freeze-dried powder also hold true for spray dried powder. Additionally, spray drying is considerably less expensive to run than the former. Whilst it has been successfully applied in this context, the relatively high temperatures required for drying cycles may render it incompatible with many bacteriophage species. 

### 2.4. Liposomes

Liposomes have been used to encapsulate a wide variety of substances, including hydrophilic and hydrophobic drugs, proteins, living cells, nanoparticles, quantum dots and plasmid DNAs [68]. Like cell membranes, liposomes vesicles are composed of phospholipid bilayers [69]. The hydrophobic and hydrophilic forces occurring throughout liposomes and cell membranes drive them to fuse readily on contact with one another, allowing for a facilitated means of substance delivery. This, coupled with the protection provided to the encapsulated substance against adverse external factors has led to many successful therapeutic applications and clinical trials [26]. Several procedures for producing liposomes have been described, including conventional methods such as the Bangham method, which involves reverse phase evaporation and phospholipid injection, as well as more novel approaches such as microhydrodynamic focusing [70]. Size, charge and fluidity of liposomes produced directly affect their ability to encapsulate and release a given substance [71]. 

Liposome encapsulation has been demonstrated to be a viable option for both bacteriophage stabilization as well as the delivery of phage therapeutics [71,72,73,74]. The fact that liposomes can fuse readily with cells they come into contact with broadens their scope of applications by allowing for the potential to target intracellular pathogens. This has been demonstrated by Singla et al. (2016), who encapsulated bacteriophage KPO12 into liposomes with a view to delivering the phage into macrophage cells infected with *Klebsiella pneuoniae* [74]. Working with a mouse model, the authors reported 100% protection of encapsulated phages against anti-phage antibodies in extracted mouse serum, compared to free phages which did not remain viable for more than three hours following exposure. Differences in encapsulation efficiency have been recorded between bacteriophages. Cinquierrui et al. (2018) carried out liposome encapsulation for T3 podovirus as well as phage K (myovirus) in liposomes measuring up to 300 nm in size [72]. While the titer recorded for T3 phage was 10^9^ PFU/mL after encapsulation, that of the latter was determined to be 10^5^ PFU/mL. This was attributed to interactions arising between phage K’s capsid and the liposome phospholipids. This may have resulted in phages binding to the liposome exterior, obscuring their tail fibers necessary for adsorption onto host cells. It is therefore likely that amino acid constitution of the phage capsids effects their ability to encapsulate within liposomes. This represents a potential drawback of the approach, with liposome encapsulation best considered on a phage-by-phage basis. 

The increased protection afforded to bacteriophages by liposomes also increases their retention time in vivo. This was demonstrated by Chadha et al. (2017), who also studied infections of *K. pneumoniae* using a burn wound model in mice, in which bacterial loads were found to reduce to a greater extent in mouse blood and organs following administration of a phage cocktail in liposomes as opposed to free phage [73]. The effectiveness of the former was further confirmed by measuring mortality outcomes in mice, where all animals treated with the liposome formulation survived and none survived after free phage treatment. This was found to be case even when the treatment was delayed by 24 h. While liposomes have demonstrated effective protection of phages in animal blood, the high acidity conditions encountered when treating gastrointestinal infections present a greater challenge, as demonstrated by Colom et al. (2015) [75]. In their work, bacteriophages UAB_Phi20, UAB_Phi78, and UAB_Phi87 targeting *Salmonella* strains were encapsulated in positively charged (between +31.6–35.1 mV) liposomes ranging from 309 to 326 nm in diameter. The liposomes were then subjected to gastric fluid conditions (pH 2.8) in which phage titers dropped 3.7 to 5.4 logs. Despite this notable drop, liposomes still performed better than free phage, for which 5.7 to 7.8 log drops were recorded. In subsequent treatment of *Salmonella*-infected broiler chickens with the liposome and liquid phage formulations, both were able to provide protection to the animals when administered daily, however with encapsulated phages protection remained for up to 1 week after treatment was stopped, by which time all activity of the non-encapsulated phages had disappeared. 

As with emulsion-based formulations, the problems that could arise with respect to long-term stability of liposome-encapsulated phage need to be considered. The potential requirement for refrigeration or post-production processing would add to the overall production costs and the liquid nature of the final product would make transportation and storage more challenging. As has been demonstrated in numerous in vivo studies, the main benefits of liposome encapsulation draw from improved delivery of phage to target cells as well increased protection afforded to encapsulated phages against adverse in vivo conditions.

### 2.5. Electrospinning

The production of nanofibers through electrospinning can be used to stabilize bacteriophages and produce antibacterial fibers [76]. For this technique, a charged, molten polymer solution is drawn onto an electrode of opposite charge. The final, dried nanofibers typically measure 100 nm or less in diameter [76]. The addition of bacteriophages to the liquid polymer prior to carrying out the process results in encapsulated bacteriophages within the nanofibers, which can be applied for both water-soluble and insoluble polymers. 

The electrospinning process does present challenges to bacteriophage stability, with the high voltages used during the process resulting in a large proportion of the phages dying [77]. Furthermore, rapid evaporation of water and subsequent osmotic change in the environment surrounding electrospun phages has been identified as a cause of the low storage viability observed in some studies [77,78,79]. Viability during the electrospinning process as well as during subsequent storage may be improved by adding magnesium salts and excipients such as trehalose [80]. Despite these observations, Diaz et al. (2018) demonstrated the applicability of the electrospinning process to a broad range of bacteriophages by integrating Fersis and PhageStaph commercial phage cocktails into nanofibers formed from a soluble (polyethylene glycol) and biodegradable polymer (polyester urea) [81]. Viable phage titers were broadly conserved across samples, with a slight decrease in the case of polyester urea fibers when lyophilized phage was used instead of the original solutions, and with the resultant nanofibers demonstrating antimicrobial activity against corresponding bacterial hosts, inhibiting growth for up to 80 h after exposure. The authors suggested that phages can survive exposure to electric fields up to 40 kVcm^−1^ for 5 min. These findings agree with observations made by Andriolo et al. (2018) who found that both electric field voltage and solvent had a negligible effect on phage viability [82]. Heat exposure, on the other hand, did adversely affect phages, with temperatures exceeding 55 °C resulting in complete loss of phage viability. It is advisable to use polymers with melting points falling below this value, however, much like spray drying, the higher temperatures associated with this process could make it challenging to avoid significant titer loss for certain bacteriophages.

Electrospinning is unique amongst encapsulation methods in that a final product can take several forms. Fibers can be broken down to form small powders, or molded into specific shapes. This could be particularly relevant to the medical devices field. Furthermore, the process can act as a bridge between encapsulation and immobilization through the act of electrospinning encapsulated phage over substrate surfaces. 

## 3. Bacteriophage Immobilization 

Immobilization refers to the chemical, physio-chemical or electrostatic binding of bacteriophages to a surface. Most research in the field of phage immobilization has been carried out for the development of pathogen biosensors, as well as for the production of antibacterial food packaging [83,84,85,86,87,88]. Despite the limited number of studies examining the use of immobilized phage for therapeutic applications, it should be considered a credible alternative to the approaches described previously, owing to the success that immobilized phage have shown in killing their bacterial targets in other applications, as well as its relative simplicity compared to some of the other approaches. Despite this, the advantages and drawbacks of the various immobilization techniques that have been described would need to be considered for this application (Table 3 and Table 4). As with encapsulation, immobilized phage can potentially be integrated into various formulations including powders, patches, wound dressings and creams, however the fact that it can technically be carried out on most surfaces increases the potential range of applications. 

Immobilized phages are stabilized through the interactions that occur between the virions and the surface. These come in the form of non-specific binding, as well as more permanent covalent bonds [23]. A key difference between immobilization and the encapsulation-based approaches discussed previously is the ultimate location of the phages, as immobilized phages are usually exposed to the external environment. While this has implications with respect to stability, it also allows for more direct contact between the phage and the target bacteria.

Immobilized phages can be rigidly attached to the surface, so the spatial orientation of the phages post immobilization is a factor that needs to be considered. In the case of tailed phages, a ‘tail-up’ orientation is very much preferred to facilitate binding to bacteria and DNA injection, with evidence suggesting that controlling orientation can drastically increase the concentration of infective immobilized phage [86,92]. This does not apply to phages such as PRD1 and PR772, in which receptor binding sites are uniformly distributed on their capsids [93]. Another factor that affects the ability of immobilized phages to infect their target bacteria is coating density and efficiency. Being able to consistently apply phages in a uniform density over the surface is desirable, as it allows for reproducibility, as well as the fact that phage clustering and non-uniform immobilization has been observed to hinder efficient bacteriophage function [94]. 

### 3.1. Physical Adsorption

Physical adsorption, or physisorption, refers to the adhesion of particles onto a surface brought about by van der Waal’s forces, dipole-dipole moments, electrostatic forces and steric and hydrophobic interactions [95]. Van der Waals forces, although weak, occur in all molecular species. This makes physical adsorption a ubiquitous occurrence. It represents a quick and relatively simple ways to immobilize a species onto a given surface and, because it occurs through non-chemical interactions, it typically does not result in any chemical alteration of the absorbate. However, because of these physical stresses as well as extremes of acidity, temperature and ionic strength can act to reduce attachment or reverse it post immobilization [23]. For example, Singh et al. (2009) found the density of immobilized phage to decrease by 8 phages/μ^2^ when the temperature was lowered from 40 °C to ambient [89]. 

Unaided physical adsorption is a term used to describe the direct application of the adsorbent to the surface. The simplicity of this approach means that complex preparation steps are avoided, with the procedure typically involving the exposure of the surface in question to a high concentration solution of the absorbate. Bennett et al. (1997) studied the unaided adsorption of phages during the development of a technique for the separation of pathogenic *Salmonella* strains from foodstuffs [96]. Physisorption of the lytic phage Sapphire was achieved by exposing polystyrene strips to high concentration phage solutions and incubating overnight. Similarly, *E. coli* biosensors have been produced following the immersion of long-period fibres in bacteriophage T4 lysate [97]. Unaided physical adsorption is often used as a control when testing out alternative immobilization strategies [89,90,94]. 

The use of bandages soaked in bacteriophage lysate to treat topical infections can be considered a form of unaided physical adsorption. Abul–Hassan et al. (1990) made use of such a strategy to treat burn wound sepsis caused by *Pseudomonas aeruginosa* in 30 patients [98]. Dressings containing adsorbed phage were applied to infected wounds, with positive effects observed in 24 of the patients. Similarly, Kifelew et al. (2020) showed that gauze soaked in purified phage cocktail AB-SA01 was effective in decreasing bacterial load of multidrug resistant *Staphylococcus aureus* and promoting diabetic wound closure in mice [99]. Application of bacteriophages in this manner might work effectively in cases of immediate application; however, it is unlikely that wound dressing and other medical devices soaked in phage lysates can be stored long term without significant reduction in phage titer and, consequently, overall efficacy. 

A drawback of unaided physical adsorption is the fact that the process is chemically undirected. The reliance on weaker, random interactions occurring between the phage and surface leaves limited opportunity to direct the process and spatially arrange the attached phage. In most cases, physical adsorption leads to undesirable disorganized attachment, with adsorbed particles bound in all orientations and with non-uniform spacing and aggregation a common occurrence [100]. The Langmuir–Blodgett technique has been used as a means of depositing bacteriophages as a monolayer on various substrates [92,101,102,103]. This increases uniform spacing between particles, which facilitates the accurate quantification of bound phage and increases targeting efficiency. The organization of phages into single layers could also be advantageous during commercial scale up, as a means of reducing phage wastage and decreasing production costs. 

Aided physical adsorption refers to techniques that actively promote the interactions that give rise to physical binding. Dipole-dipole and hydrophobic interactions, as well as electrostatic forces, represent the strongest types of non-covalent binding that occur between a surface and absorbate [95,104]. In the case of hydrophobicity, the occurrence of both polar and non-polar amino acid residues makes it difficult to reliably predict immobilization efficiency, outside of empirical testing. This is illustrated by the contradictory results obtained when measuring the hydrophobicity of MS2 bacteriophage in two separate studies, despite both using the same hydrophobicity assay [105,106,107]. MS2 is one of the only phages tested for its ability to adsorb to surfaces using hydrophobic interactions [104,108,109,110]. One reason for this could be fears that hydrophobic interactions can disrupt protein function by encouraging unfolding, or alternative folding arrangements, a phenomenon that has been reported for the hydrophobic-mediated immobilization of enzymes [111]. Just as hydrophobic bonds have high affinity for each other, the same can be said of regions of high polarity, which can give rise to dipole-dipole interactions. In covalent bonds between atoms of different electronegativities, the electron cloud is distributed unevenly, with either atom assuming a partial positive or negative charge (a dipole) [112]. Opposite partial charges from different covalent bonds are attracted to each other, bringing about dipole-dipole interactions. This phenomenon can be utilized in phage immobilization. Here, polar amino acid sidechains on the surface of the capsid are made to form dipole-dipole interactions with a polar activating layer deposited on the substrate. Singh et al. (2009) improved the immobilization efficiency of T4 on gold surfaces by applying layers of sugars and amino acid coatings, resulting in up to a 7-fold increase in bacteriophage adsorption [89]. Similar loading increases have been observed on gold surfaces with *S. aureus* phages [113]. Adsorption has also been enhanced through addition of phage to printing ink formulations, with polar molecules in the ink resulting in phage retention on the surface following printing [114,115]. Such an approach demonstrates aspects of both immobilization and encapsulation. 

### 3.2. Charge-Directed Immobilization

In charge-directed immobilization, electrostatic attraction between permanent opposing charges on the surface and adsorbent is used to bring about immobilization. These are considerably stronger than the interactions discussed thus far. Bacteriophages often consist of charged regions; phage heads usually possess a net negative charge with the opposite being true for their tails [60,104]. For this reason, phages have been shown to bind tail-down or tail-up, depending on the net charge present on the surface [116]. It follows that the application of positive charges to surfaces is a favored strategy to bind phages in the desired tail-up orientation. Anany et al. (2011) carried out charge-based immobilization of phage cocktails targeting *E. coli* and *Listeria* host strains. Phage cocktails of different concentrations were applied to cellulose disks pre-treated with 0.5% wt/vol polyvinylamine polymer. It was concluded that the net positive charge on treated surfaces lead to increased immobilization efficiency [60]. Similar results with positively charged substrates have been reported in other studies [90,116,117]. Comparative studies have shown more phage binding in charge-based immobilization, when compared to other reversible approaches, which is likely due to the strength of the interactions [90]. This is supported by the observation that it has been shown to perform less efficiently than covalent-based immobilization, which results in an even stronger interaction [82]. The technique can be applied in conjunction with other immobilization approaches as a means of guaranteeing the desired bacteriophage orientation with enhanced attachment strength [118]. A recent study found that the application of alternating current across a gold surface functionalized with polar molecules resulted in a dense, ordered layer of phages in tail-up conformation [91]. 

As with physical adsorption, electrostatic binding is also influenced by physiochemical properties of the medium, such as ionic strength and pH, which can directly affect protein charge through the isoelectric effect. This was demonstrated by Peng et al. (2011), who increased the pH level in the surrounding medium beyond the isoelectric point of tobacco mosaic virus [119]. The negative charge on the phage heads was increased, leading to increased tail-up orientation on gold surfaces. It is therefore recommended that the isoelectric point of the phage is known prior to carrying out charge-based immobilization, to allow targeted process optimization. The fact that most phage heads possess net negative charges makes this approach broadly applicable across most phage groups. Despite this, one limitation is the resulting attraction/repulsion-based forces that may arise between treated materials. In the case of polymer sheets for example, cationic surfaces may bind to the uncharged side of sheets packed on top of them, making for difficult handling post-production.

### 3.3. Protein Ligand

The natural tendencies of proteins to adsorb to certain ligands can be exploited for the purpose of bacteriophage immobilization. The surface and absorbate are coupled with a binding protein and its corresponding ligand, respectively, with the interaction and subsequent immobilization occurring once they encounter one another. Streptavidin is a protein that occurs in the bacterium *Streptomyces avidinii* [120]. It has a strong affinity for biotin, a vitamin involved in several metabolic processes, and binds to it through one of the strongest non-covalent interactions known [121]. Protein-ligand interactions like this have been used for bacteriophage immobilization [86,92,116,122,123,124]. Ligands such as biotin are normally crosslinked to bacteriophages through ester activation with N-hydroxysuccinimide (NHS) and other carboimmides [86,92,122,125]. NHS-biotin reacts with primary amines found in side-chains of amino acids such as lysine, as well as terminal amino groups of polypeptides, resulting in the permanent attachment of biotin. This covalent process is known as biotinylation and there are numerous examples of it being carried out with enzymes [126,127]. Alternatively, the gene coding for the ligand or binding protein of interest can be integrated with the bacteriophage genome. Tolba et al. (2010) fused the genes bccp and cbm, which code for biotin carboxyl carrier protein and cellulose binding domain respectively, with the soc gene of T4 phage [86]. The protein coded for by soc, small outer capsid protein, forms part of the phage capsid, resulting in the expression of biotin carboxyl carrier protein or cellulose binding protein on the T4 head which permitted subsequent affinity immobilization onto respective streptavidin and cellulose-containing surfaces. Coupling a phage with a binding agent this way allows for the strategic positioning of the later. This is especially relevant to tailed phage immobilization, in which the ‘tail-up’ orientation is desired. The major downside to this approach is the complexity of the procedure, which requires a relatively detailed knowledge of the phage, and significant time and resources to plan and effectively execute immobilization. In addition to this, altering a phage’s genome can potentially result in undesired changes to its activity, such as a decreased burst size and an extended latency period [86]. Bacteriophage attachment using protein-ligand interactions has been shown to be effective, with Gervais et al. (2007) reporting on a 15-fold increase in phage binding compared to unaided physisorption [122]. 

### 3.4. Covalent 

All of the approaches discussed so far have not involved the alteration of substances through the formation of new chemical bonds. Covalent immobilization represents the most permanent and irreversible form of attachment, demonstrated by the ability of covalently immobilized phages to remain bound to substrate even after prolonged exposure to sonication forces [128]. The ability to withstand mechanical stresses could play a role in future development of robust therapeutic products. Studies have also found covalent-based approaches allow for increased binding efficiency, with one study reporting a 37-fold increase in binding efficiency compared to unaided physical adsorption [89,100]. Covalent immobilization can be achieved by crosslinking phage to the substrate. Bacteriophages react covalently through amino acid residues protruding from their viral capsids. These include carboxylic groups from glutamine and aspartic acid, amines from lysine, sulfide groups from cysteine and phenols from tyrosine [129]. In some cases, phages have been observed to bond on mere exposure to certain substrates. M13 phage, for example, readily binds to sulfur particles covalently through carboxylic acid functional groups on glutamine and aspartic acid residues [130]. The technique employed here is similar to the biotinylation technique described previously except in this case, the phage is cross-linked directly to the surface as opposed to an affinity-binding protein. As with protein-ligand immobilization, carboiimide-based cross-linking is often favored as a means of bioconjugation in covalent-based immobilization [82,91,93,128,131]. Janczuk et al. (2017) used EDC to activate carboxylic groups on magnetic fluorescent beads. Subsequent phage attachment via lysine residues resulted in the formation of amide linkages to EDC. Non-carbodiimide-based cross-linkers that have been applied to bacteriophage immobilization include glutaraldehyde and maleic anhydride [89,132]. The main drawbacks to linker-based covalent attachment arise from the potential disruptions to phage activity after bond formation. If a bonding occurs with a residue near the phage adsorption site, this can potentially obscure it, limiting or eliminating the ability of the phage to bind to its target. Additionally, the process is relatively complicated, and could present significant challenges with respect to scale-up.

The direct covalent binding of bacteriophages to surfaces in the absence of cross-linkers has also been reported [133]. Here, the authors attached phage vB_Pae_Kakheti25 onto polycaprolactone fibers. These were subjected to acidic conditions for activation prior to bacteriophage attachment. Effective killing of the host was observed even after 25 rinses of the substrate, demonstrating the robustness of the attachment. Simpler processes such as the one described are appealing as a means of producing bioactive surface on a large scale, due to the lower costs associated with the process. An area of increasing interest concerns the use of plasma treatment to achieve immobilization. 

The ionisation of gaseous particles through electron bombardment results in the formation of plasma [134]. Gas in this state typically consists of a mixture of ionized particles, free electrons and radicals, whose application results in chemical changes to treated surfaces [135]. These can be exploited to permanently coat surfaces with substances of interest. Initial studies into plasma-mediated immobilization found that it allowed for substances to be deposited consistently, resulting in production of layers of uniform thickness and limited damage to the adsorbate [136]. In their covalent immobilization of phages T1 and ɸ11, Pearson et al. (2013) first grafted maleic anhydride onto polyethylene and polytetrafluoroethylene surface using microwave plasma [132]. This acted as a linker to allow amide bonding with amine residues on the phages. Wang et al. (2016) used reactive ion etching on polyhydroxyalkanoate surfaces for EDC/sulfo-NHS linker-based immobilization of T4 phage [137]. The linker was attached to the surface either through reaction with plasma generated carboxylate groups on the surface, or through graft polymerisation of acrylic acid with subsequent addition of the linker. T4 bacteriophage was then covalently bound to the surface. The authors of this study compared immobilization efficiency of plasma-treatment with and without linkers. An interesting observation was the higher efficiencies for immobilization performed in the absence of linkers. Considering the increased costs and complications of using linkers for the attachment of biological entities, this suggests that plasma-based techniques may have an important role to play in developing stable formulations for therapeutic phage applications. Linker-free immobilization is believed to occur through direct reaction of the adsorbate with free radicals generated on the treated surface, resulting in covalent bond formation [138]. Therefore, conditions which stabilize radicals on the surface, increase the efficiency of covalent bond formation. Tropoelastin was covalently immobilized onto polyethersulfone (PES) treated with plasma-immersion ion implantation, yielding permanent biofunctionality to the material [139]. 

Plasma-based immobilization of bacteriophages has already started being commercialized. At Fixed Phage Ltd (Glasgow, UK), corona discharge is used to bind bacteriophages covalently to a range of substrates, such as food packaging, wound dressings, animal feed and powders, for formulation into creams and gels [140,141]. In agreement with other studies, promising results have been generated demonstrating substantially enhanced phage stability after immobilization. For example, bacteriophages specific for *Vibrio parahaemolyticus* were immobilized onto shrimp feed and shown to retain titers sufficient to treat disease for more than 250 days after storage at 30 °C [142]. and manuscript in preparation. In the same study, phages applied to feed without prior corona treatment lost all activity after 21 days. In a subsequent tank trial, phage-treated feed was shown to protect *Thor amboinensis* model shrimps against a *V. parahaemolyticus* challenge [143]. 5 days following exposure to the pathogen, 80% of shrimps receiving regular feed died whilst 90% of the phage-treated group were still alive. The technology has also been applied to demonstrate extensions of shelf life in bagged spinach. A phage cocktail targeting *Pseudomonas* was developed and immobilized onto plastic inserts. Bags containing the inserts demonstrated a 1 day increase in shelf life compared to their untreated counterparts [manuscript in preparation]. Corona discharge is a well-established industrial process, is relatively cheap to operate and can be applied onto most materials to activate them. These factors make it, along with other plasma-based immobilization processes, ideally suited for use in formulating effective, stable therapeutic phage formulations. 

## 4. Conclusions and Future Prospects

Bacteriophages represent a viable treatment alternative for bacterial-borne diseases. Their application in clinical settings as a last resort treatment has demonstrated their potential in individual patients but for the widespread therapeutic use of licensed phage products to be achieved in the future, two conditions will need to be satisfied: the successful completion of clinical trials proving their efficacy in a significant portion of cases, and an economically and qualitatively viable means of mass production.

The application of specialized formulations will be key to any future clinical trial successes in bacteriophage therapy. The results of recent studies strongly suggest that phage formulations can act to stabilize phages against adverse in vivo conditions while also offering a more pragmatic route of administration compared to liquid phage preparations. While substantial progress has been made with encapsulation-based approaches, another promising approach to the formulation of phage therapeutics is through bacteriophage immobilization. Bacteriophage diversity can affect the extent to which a given formulation process will be successful, emphasizing the importance of selecting the most appropriate approach for the phage(s) being considered. This has increased the need for a comparative analysis of the different strategies currently available and better understanding of the role phage diversity plays in this regard. Another point of consideration is the potential variability of pathogenic strains across patients, which will require the stable and cost-effective formulation of large phage cocktails offering maximum coverage to offset any differences.

In addition to the other considerations, formulations will need to be considered in terms of the ease at which their production can be scaled up. In cases such as linker-based immobilization, multiple processing steps would be involved, while higher running cost and increased lead times would be associated with other processes such as freeze-drying. The most attractive phage products from a production point of view will therefore be those that are relatively straightforward to produce consistently. Formulations which can be stored for extended periods of time at ambient temperatures are also more likely to be favored.

It is likely that different formulation methods will be required for different applications and that further research is needed in this area to facilitate the widespread use of phages as genuine viable alternatives to other antibiotics in human therapy.

## Figures and Tables

**Table 1:** Examples of the various encapsulation approaches that have been carried out with bacteriophages.

Encapsulation Method	Bacteriophage (Host Genus)	Formulation	Observations	Reference
Emulsification	K (*Staphylococcus*)	Semi-solid	Up to 10 days of activity at 20 °C	[27]
Freeze-Drying	M13 (*Escherichia*)	Powder	<1 log drop in titer after 2 months at ambient temperature	[41]
Spray-Drying	PEV2, PEV40 (*Pseudomonas*)	Powder	<1 log drop in titer after 1 year at 20 °C	[39]
Liposome Entrapment	KP01K2 (*Klebsiella*)	Liquid	Up to 14 days of activity in vivo	[42]
Electrospinning	Felix O1 (*Salmonella*)	Nanofibers	Phage activity of equivalent to 10^5^–10^6^ PFU/mL after fiber preparation	[43]

**Table 2:** Summary of the benefits and limitations associated with the mass production of encapsulated therapeutic phage formulations.

Encapsulation Method	Benefits	Limitations
Emulsification	Material produced ideal for cream-type treatments Promote absorption when applied topically	Difficult to transport/store at large scale Prone to bacterial contamination Only stable when refrigerated
Freeze-Drying	Final product easy to store/transport High stability post-production Variety of applications	Time-consuming, costly process Ice crystal formation can decrease phage viability
Spray-Drying	Final product easy to store/transport High stability post-production Variety of applications	Energy-consuming process Temperature can decrease phage viability during process
Liposome Entrapment	Protection of phages against in vivo conditions Extensive studies demonstrating therapeutic effect compared free phage	Encapsulation yield of phages in liposomes difficult to control Difficult to transport/store at large scaleOnly stable when refrigerated
Electrospinning	Diverse array of materials can be produced. Easy deposition of fiber-encapsulated phage onto other substrates	Fiber-spinning process can damage phages

**Table 3:** Examples of studies involving immobilization of bacteriophages onto surfaces.

Immobilization Approach	Bacteriophage (Host Genus)	Surface	Observations	Reference
Physical Adsorption	T4 (*Escherichia*)	Gold surface modified with cysteine and glutaraldehyde	Phage surface concentration of 18 ± 0.15 phages per um^2^	[89]
Protein-Ligand	T4 (*Escherichia)*	Magnetic beads, microcrystalline cellulose beads	Up to 81% improved binding efficiency compared to physical adsorption	[86]
Electrostatic	T7 (*Escherichia*)	Cellulose microfibers	15–25% phage loading efficiency on surface	[90]
Covalent Linkage	AG10 (*Escherichia*) CG4 (*Salmonella*)	Magnetic-fluorescent beads	Phage activity equivalent to 10^8^ PFU/mL observed in material	[91]

**Table 4:** Summary of benefits and limitations associated with various bacteriophage immobilization techniques for the production of therapeutic phage formulations.

Immobilization Approach	Benefits	Limitations
Physical Adsorption	Simple process Inexpensive	Undirected, inconsistent Phage not strongly bound to substrate
Protein-Ligand	Strongly bound phage High binding efficiency Tail-up orientation	Complicated process Expensive
Electrostatic	High binding efficiency Applicable to most tailed phages Tail-up Orientation	Electrostatically charged surface may not be desirable
Covalent Linkage	Strongly bound phage Potentially longer shelf life	Can be a costly and complex process (in the case of linker-based immobilization)

## Data Availability

Not applicable.
